# Quantification of tumor fluorescence during intraoperative optical cancer imaging

**DOI:** 10.1038/srep16208

**Published:** 2015-11-13

**Authors:** Ryan P. Judy, Jane J. Keating, Elizabeth M. DeJesus, Jack X. Jiang, Olugbenga T. Okusanya, Shuming Nie, David E. Holt, Sean P. Arlauckas, Phillip S. Low, E. James Delikatny, Sunil Singhal

**Affiliations:** 1 University of Pennsylvania Perelman School of Medicine, Department of Surgery, Philadelphia, 19104, United States of America; 2 Emory University, Departments of Biomedical Engineering and Chemistry, Atlanta, 30322, United States of America; 3 University of Pennsylvania School of Veterinary Medicine, Department of Clinical Studies, Philadelphia, 19104, United States of America; 4 Perelman School of Medicine at the University of Pennsylvania, Department of Radiology, Philadelphia, 19104, United States of America; 5 Purdue University, Department of Chemistry, West Lafayette, 47907, United States of America

## Abstract

Intraoperative optical cancer imaging is an emerging technology in which surgeons employ fluorophores to visualize tumors, identify tumor-positive margins and lymph nodes containing metastases. This study compares instrumentation to measure tumor fluorescence. Three imaging systems (Spectropen, Glomax, Flocam) measured and quantified fluorescent signal-to-background ratios (SBR) *in vitro*, murine xenografts, tissue phantoms and clinically. Evaluation criteria included the detection of small changes in fluorescence, sensitivity of signal detection at increasing depths and practicality of use. *In vitro*, spectroscopy was superior in detecting incremental differences in fluorescence than luminescence and digital imaging (Ln[SBR] = 6.8 ± 0.6, 2.4 ± 0.3, 2.6 ± 0.1, p = 0.0001). In fluorescent tumor cells, digital imaging measured higher SBRs than luminescence (6.1 ± 0.2 vs. 4.3 ± 0.4, p = 0.001). Spectroscopy was more sensitive than luminometry and digital imaging in identifying murine tumor fluorescence (SBR = 41.7 ± 11.5, 5.1 ± 1.8, 4.1 ± 0.9, p = 0.0001), and more sensitive than digital imaging at detecting fluorescence at increasing depths (SBR = 7.0 ± 3.4 vs. 2.4 ± 0.5, p = 0.03). Lastly, digital imaging was the most practical and least time-consuming. All methods detected incremental differences in fluorescence. Spectroscopy was the most sensitive for small changes in fluorescence. Digital imaging was the most practical considering its wide field of view, background noise filtering capability, and sensitivity to increasing depth.

Several groups have recently developed optical imaging strategies for clinical surgery using injectable fluorescent contrast agents to identify tumors intraoperatively^1^,^2^,^3^. Current fluorophores include non-specific contrast agents, such as indocyanine green (ICG)^2^,^4^ and 5-aminolevulinic acid (5-ALA), and receptor-targeted tumor-specific dyes, such as folate-fluorescein (EC-17)^3^. Intraoperative tumor fluorescence allows surgeons to identify small tumors^2^, margins^1^, lymph nodes^5^, and metastatic disease^4^. In many clinical scenarios, however, tumors have low affinity for these tracers and autofluorescence from normal tissue can cause difficulties in identifying small areas of cancer cells. Thus, one goal of optical imaging instrumentation is to optimize the fluorescence data that is available in order to make intraoperative decisions.

The objective of this study was to perform a preclinical comparison of three technologies that can be used for optical imaging: spectroscopy, luminometry, and digital imaging. Spectroscopy is based on optical fibers that can sample tissues locally (typically 1 mm^3^ volume). Spectroscopic devices can be compact allowing light delivery and collection in close proximity. Several groups have previously described spectroscopic devices to quantify fluorescent signal from solid tumors^6^,^7^,^8^,^9^,^10^,^11^,^12^. Luminometers are photodiode detectors that can measure photoluminescence as a result of singlet–singlet electronic relaxation. Luminometers have the benefit of a wide range of detection wavelengths for measuring fluorescence. Digital imaging is based on intensified charge-coupled devices (CCD). Quantifying data from digital imaging relies on region of interest (ROI) software that converts pixel counts from fluorescent data into binary values and provides a ratio of tumor compared to background tissues^13^,^14^,^15^,^16^,^17^,^18^.

We judged each imaging modality based on three criteria. First, we identified the system providing the best sensitivity for detection of small quantities of fluorescent tissue. One of the major goals of intraoperative imaging is to locate residual tumor cells at the margins and wound bed after surgery so we compared the ability of all three systems to detect minimal fluorescence. Second, many tumors are located deep in solid organs. We hypothesized that although an imaging system may have excellent resolution, it may fail to detect fluorescence from tumors in deep tissues due to scattering and absorption. Therefore, we considered the sensitivity for each system at increasing tissue depths using a tissue phantom. Third, we examined the practicality of each system. For patient safety concerns, it is not feasible to prolong an operation excessively for the benefit of intraoperative imaging. Therefore, the ideal system for surgical application is technically easy to handle, obtains real-time data and does not require significant data processing.

## Materials and Methods

### Cell Lines

The murine lung cancer cell line, TC1, was derived from primary lung epithelial cells from C57BL/6 mice and transformed with the c-Ha-ras oncogene^19^,^20^. It was kindly provided by Steven Albelda, M.D., University of Pennsylvania. The murine Lewis Lung Carcinoma (LLC) is a non-small cell lung cancer that was obtained from the American Type Culture Collection. The human cervical carcinoma, KB, was established through HeLa cell contamination and has been previously described^21^. It was a generous gift from Steven Albelda, M.D., University of Pennsylvania. The human renal clear cell carcinoma, RCC10, has a mutated von Hippel-Lindau tumor suppressor gene and was kindly provided by Celeste Simon Ph.D., University of Pennsylvania^22^. The human ovarian adenocarcinoma cell line, IGROV-1, was isolated from a 47 year-old woman and is both drug resistant and hormone receptor negative. It was kindly provided by Janos Tanyi, M.D., Ph.D., University of Pennsylvania^23^.

TC1, RCC10, KB and IGROV-1 cell lines were cultured and maintained in RPMI (RPMI 1640 Medium; Gibco) supplemented with 10% fetal bovine serum (FBS; Hyclone), 1% penicillin/1% streptomycin and 1% glutamine. The LLC cell line was cultured and maintained in Dulbecco’s Modified Eagle Medium (DMEM; Gibco) supplemented with 10% FBS, 1% penicillin/1% streptomycin and 1% glutamine. Cell lines were regularly tested and maintained negative for *Mycoplasma spp*. using the Lonza MycoAlert™ Mycoplasma Detection Kit.

### Mice

Female C57BL/6 mice were purchased from Jackson Laboratories and female NOD.Cg-*Prkdc*^*scid*^ *Il2rg*^*tm1Wjl*^/SzJ were bred at the CHOP Barrier at the Colket Translational Research Building at the Children’s Hospital of Philadelphia. The mice were maintained in conditions approved by the Animal Care and Use Committees of the Children’s Hospital of Philadelphia and the University of Pennsylvania and in agreement with the Guide for the Care and Use of Laboratory Animals.

### Reagents

Pharmaceutical grade indocyanine green (ICG) was purchased from Akorn, Inc. (IC-GREEN, NDC 17478-701-25). Animals were injected intravenously with 5.0 mg/kg 24 hours before imaging. Pharmaceutical grade EC-17 was kindly provided by On Target Laboratories, LLC (West Lafayette, IN). Animals were injected i.v. with 0.1 mg/kg 4 hours before imaging. For *in vitro* studies, serial dilutions were created in Dulbecco’s Phosphate-Buffered Saline (PBS) from Corning (21-031-CV).

### Near-infrared and Fluorescence Imaging Platforms

The Spectropen is a home built handheld NIR imaging system, which has previously been described in detail^6^. This fiber-optic spectroscopic system uses a Raman Probe detector connected to two fiber optic cables, one for laser excitation at 785 nm and the other for light collection. This integrated spectrometer and sampling head has a wavelength range 800–930 nm with 0.6 nm spectral resolution for fluorescent measurements.

The Glomax Multi Detection System (Promega®, Madison, WI) was used in fluorimeter operation mode to quantify EC-17 fluorescence from samples placed into 96-well microplates. Wavelength matched LEDs provide the excitation light. The blue wavelength snap-in optical kit excites at 490 nm and has a detection range of 510-570 nm. A PIN-photodiode top-reads the amount of emission. The SpectraMax® M5 Multi-Mode Microplate Reader (Molecular Devices, Sunnyvale, CA) was used to quantify NIR fluorescence. This fluorimeter uses a 50 watt xenon light source, and has a wavelength range from 250-850 nm. A photomultiplier top-reads the emission intensity. The SpectraMax® was used for all ICG quantification experiments.

The “Flocam” is a home built digital imaging system based on a dual CCD camera system previously described^13^ (BioVision Technologies Inc, Exeter, PA). The system uses two QIClick™ digital CCD cameras from QImaging (British Columbia, Canada), one for white brightfield and one for fluorescence overlay. The cameras have a 696 × 520 pixel resolution and have a fluorescence exposure time of 20–200 ms. Each camera runs on 6 W supplied through a firewire interface. The light source is a Spectra X Light Engine (Lumencor, Inc., Beavertown, OR). Six special-order NIR bandpass filters are employed to produce the excitation light. Using fluorescent images in ImageJ, the region-of-interest tool was used to quantify pixel intensity of tumor, fluorescent tracer and a background signal. Muscle or PBS was used as the background signal. SBR was generated by dividing the fluorescent signal by the background signal. Each experiment was repeated in triplicate. Because Spectropen SBRs were often an order of magnitude higher than luminometry or digital imaging SBRs, comparisons between these three were evaluated using the natural log.

### *In vitro* phantom models

For *in vit*r*o* standard curve measurements, we used black 64-well plates with serial dilutions: ICG and EC-17 ranged from 2.81 × 10^−6^ M to 7.26 × 10^−6^ M. For tissue depth penetration phantoms, rubber latex balloons were loaded with 3.23 μM ICG until they reached 1.0 cm, 2.0 cm, 3.0 cm, 4.0 cm and 5.0 cm in diameter. To mimic human adipose tissue, the tumor phantom was placed in a 1 L glass beaker and submerged in semi-solid butter at 5 mm increments between 0 and 3 cm. The spectrometer and the digital capture software quantified fluorescence of the submerged tumor phantoms at each depth. All measurements were taken at the top surface of the phantom. SBRs were generated for each sized tumor phantom, and this information was plotted against the depth of penetration.

### Murine Flank Tumor Model

Mice were injected subcutaneously in the flank with 1.2 × 10^6^ TC1 cells (C57BL/6 mice), 1.0 × 10^6^ LLC cells (C57BL/6 mice), 2.0 × 10^6^ KB cells (NOD.Cg-*Prkdc*^*scid*^ *Il2rg*^*tm1Wjl*^/SzJ mice) or 1.0 × 10^6^ IGROV-1 cells (NOD.Cg-*Prkdc*^*scid*^ *Il2rg*^*tm1Wjl*^/SzJ mice). Tumor cells for subcutaneous flank injections were suspended in 100 μL of PBS. All mice were maintained in pathogen-free conditions and used for experiments at ages 8 week or older. The Animal Care and Use Committees of the Children’s Hospital of Philadelphia and the University of Pennsylvania approved all murine protocols in compliance with the Guide for the Care and Use of Laboratory Animals (Protocol# 804894).

Once the tumors reached 500 mm^3^, mice were anesthetized with intramuscular ketamine (80 mg/kg) and xylazine (10 mg/kg), shaved, and the surgical field was prepared for aseptic surgery. After surgical resection was performed, both the tumor and tumor bed were fluorescently imaged according to a previously described model^24^. After imaging, the incision was closed using sterile silk 4-0 braided sutures (Ethicon Inc., NJ).

### Pilot Human Study

All research was approved by the Institutional Review Board (IRB) at the University of Pennsylvania and patients gave informed consent for the procedure as previously published^2^. The study was carried out in accordance with all IRB approved guidelines. In conjunction with an ongoing clinical trial (NCT02280954), patients with lung masses suspicious for pulmonary carcinomas received ICG intravenously prior to lobectomy. After resection, portions of the tumor were placed in a black 96-well plate and fluorescently imaged.

### Data analysis

For experiments comparing differences between 2 groups, one-tailed Student t-tests were used. One way Analysis of Variance (ANOVA) was used for experiments containing three sets of data. All statistics were run with an alpha level α = 0.05. Data are presented as mean, and all values after the mean are reported as standard deviations (STD). For purposes of consistency, we set data acquisition times to 30 milliseconds on all imaging devices.

## Results

### Optical contrast agents can be used for intraoperative imaging

**Figure 1:**
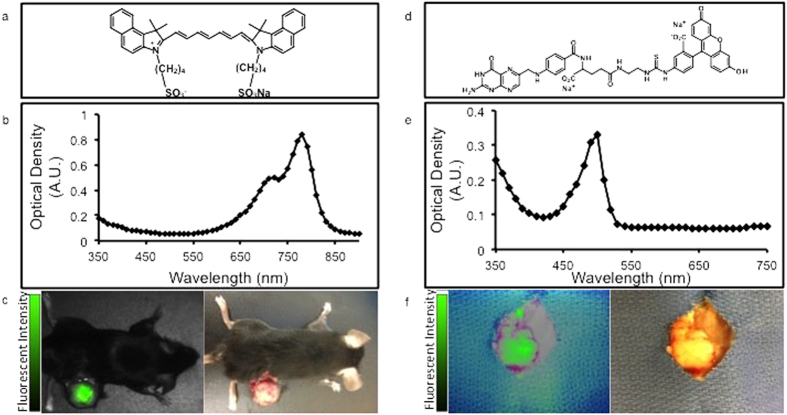
(**a**) Chemical structure of ICG. (**b**) Absorbance spectrum of ICG. Y-axis is measured in arbitrary units. (**c**) Fluorescence and bright field image of a C57BL/6 mouse bearing a LLC flank tumor injected intravenously with with ICG. (**d**) Chemical structure of EC-17. (**e**) Absorbance spectrum of EC-17. Y-axis is measured in arbitrary units. (**f**) Fluorescence and bright field image of an excised KB tumor from a C57BL/6 mouse that has been injected intravenously with EC-17.

In order to model intraoperative molecular imaging, a murine tumor model was developed to test two biocompatible fluorophores in clinical use for cancer surgery: indocyanine green (ICG) and folate-fluorescein isothiocyanate (EC-17). ICG is a water-soluble cyanine dye that was granted FDA approval in 1959 (Fig. 1a) and has multiple applications for fluorescence-guided surgery in various specialties^25^,^26^,^27^,^28^. The molecular weight of ICG is 774.96 grams/mol. It is amphiphilic, and it has a peak excitation (λ_ex_) and peak emission (λ_em_) wavelength at 778 and 832 nm, respectively (Fig. 1b). EC-17 is a folate-fluorescein isothiocyanate (folate-FITC) conjugate (Fig. 1d) with a molecular weight of 916.83 grams/mol. Its peak λ_ex_ and λ_em_ wavelengths are 494 nm and 521 nm, respectively (Fig. 1e).

C57bl/6 mice (n = 15) were injected with either the TC1 (7 mice) or LLC (8 mice) cell line subcutaneously in the right flank. NOD.Cg-*Prkdc*^*scid*^ *Il2rg*^*tm1Wjl*^/SzJ mice (n = 15) were injected with either the KB cell line (7 mice) or IGROV cell line (8 mice). Once the tumors reached 500 mm^3^, the animals were injected via tail vein with either 5 mg/kg of ICG (24 hours prior to surgery) or 0.1 mg/kg of EC-17 (4 hours prior to surgery). Once the flanks were exposed, two imaging devices (digital imaging and spectroscopy) were utilized to assist in the surgical resection of the tumor as previously described^1^,^6^,^13^,^24^.

First, the surgeon reviewed optical images from the digital camera system and subjectively decided if the tumor was fluorescent. In 15 animals that received ICG, the surgeon identified 15 out of 15 (100%) animals to have fluorescent flank tumors (Fig. 1c). In 15 animals that received EC-17, the surgeon identified 15 out of 15 (100%) animals to have fluorescent flank tumors (Fig. 1f). When asked to subjectively rank the tumors based on the degree of fluorescence, the surgeon could not identify any significant difference.

**Figure 2:**
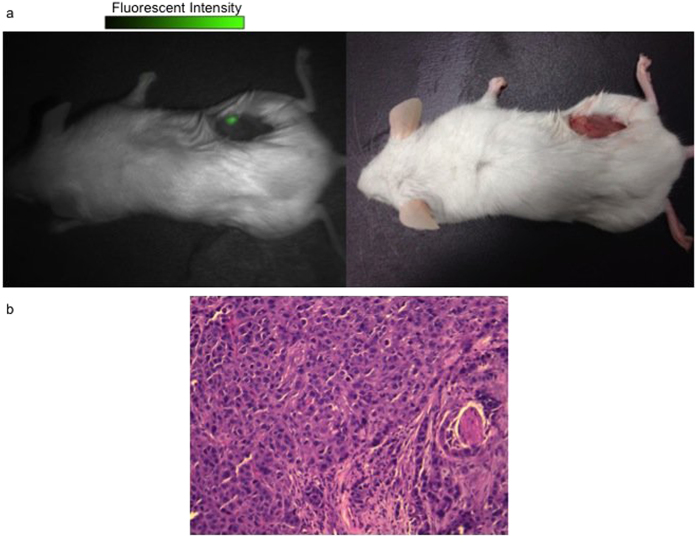
(**a**) Residual fluorescent tumor foci detected in the surgical bed by fluorescence imaging after macroscopic tumor resection. (**b**) H&E staining was performed on the tumor margin and was confirmed by a pathologist to contain tumor cells.

The surgeon then removed the tumors using standard-of-care palpation and gross visual inspection to determine tumor margins. All tumors were imaged *ex vivo* using spectroscopy, luminometry and digital imaging. In all cases, the surgeon felt the entire tumor had been removed. Then, spectroscopy and digital imaging were used to confirm that the wound margins were tumor-free *in vivo* as previously described^1^,^2^,^24^. Using both approaches, intraoperative imaging identified 3 out of 15 (20%) animals who received ICG had residual disease, which required further resection (Fig. 2a). This residual tumor tissue was removed, preserved in formalin and sectioned in paraffin blocks. It was stained with Hemotoxylin and Eosin (H&E) (Fig. 2b). Similarly, this approach discovered 2 out of 15 (13%) mice who received EC-17 to have positive margins that required further resections. All residual fluorescence was histologically confirmed to contain tumor cells by a pathologist. The wounds were surgically closed, and the animals were monitored for recurrence for 6 weeks. None of the animals recurred. In total, intraoperative imaging had 100% true positive and a 0% false positive rate for detecting fluorescence in non-cancerous tissues.

### Fine point discrimination between intraoperative imaging techniques

Although the surgeon could not differentiate the degree of fluorescence by visual inspection, current imaging technologies can provide quantitative measurements of tumor fluorescence. To identify the optimal method to quantitate signal-to-background (SBR) ratio of the tumor to the surrounding normal tissue, we compared the three intraoperative imaging technologies: spectroscopy^6^, luminescence and optical imaging^13^. Our goal was to determine which approach could provide superior sensitivity to small incremental differences in fluorophore concentrations.

**Figure 3:**
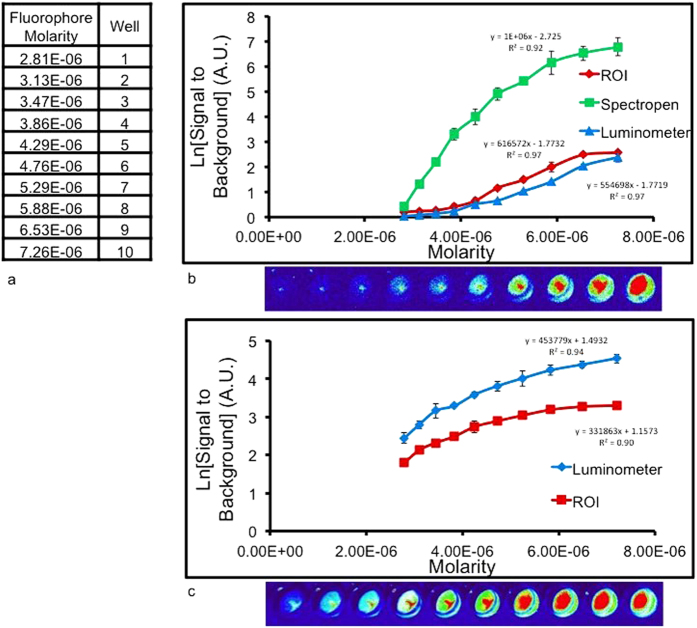
(**a**) Serial dilution concentrations of ICG and EC-17. (**b**) SBR vs ICG concentration. Y-axis is measured in arbitrary units. Error bars are reported as standard deviations (STD). (**c**) SBR vs. EC-17 concentrations. Dilutions were prepared with phosphate buffered saline. Y-axis is measured in arbitrary units. Error bars are reported as standard deviations (STD).

To standardize the quantity of ICG, ten serial dilutions (range: 7.26 × 10^−6^–2.81 × 10^−6^ M) were prepared in 96-well culture plates (Fig. 3a). The background signal was measured from a well with an equal volume of PBS. Each method (spectroscopy, luminescence, digital imaging) was used to generate a SBR ratio from each well (Fig. 3b).

From the digital images, the investigator subjectively identified fluorescence from 9 wells (range 2.81 × 10^−6^–7.26 × 10^−6^ M). Spectroscopy, luminescence and digital capture produced ln[SBR] ratios ranging from 0.4–6.8, 0.0–2.4, and 0.2–2.6, respectively (n = 10). Intra-experiment variability between replicates was small; the signal varied by 4.1% ± 4.0% in each experiment (n = 3). The SBR ratio was linear and strongly correlated with concentration for all three methods: spectroscopy, luminescence, digital imaging (r^2^ = 0.92, 0.97 and 0.97, respectively).

Spectroscopy was the most sensitive at detecting small differences in the tracer concentrations. For each 7.33 × 10^−7^ M increase in ICG concentration, the fluorescence quantified by spectroscopy changed 6,886.6 ± 924.1 arbitrary units (au). For the digital imaging system images, each 7.33 × 10^−7^ M increase in ICG, the fluorescence increased by 6.7 ± 4.2 au. For the digital imaging, for each 7.33 × 10^−7^ M increase in ICG, the fluorescence increased by 63.4 ± 2.3 au. Of note, the spectrometer generated a maximum Ln[SBR] of 6.8 for ICG, whereas the luminometer and ROI software gave a maximum Ln[SBR] of less than 3. The spectrometer’s fluorescent image sensor was saturated at the highest ICG concentration.

To evaluate EC-17, we created 10 dilutions over the same concentration range as ICG. Luminescence and digital capture techniques produced Ln[SBR] ratios ranging from 2.4–4.5 and 1.8–3.3, respectively in the 10 wells (Fig. 3c) (n = 10). Between the triplicate measurements in each technique, the signal was within 6.1% ± 4.8% of the same value, thus there was strong data fidelity across experiments. The signal was linearly correlated with EC-17 concentration using luminescence and digital imaging (r^2^ = 0.94 and 0.90, respectively).

In summary, digital imaging, luminescence and spectroscopy can quantify fluorescence. Signal intensity is directly proportional to tracer molarity. Spectroscopy appeared to be the most sensitive technique for identifying small incremental changes in fluorophore concentrations.

### Comparison of imaging techniques *in vitro*

**Figure 4:**
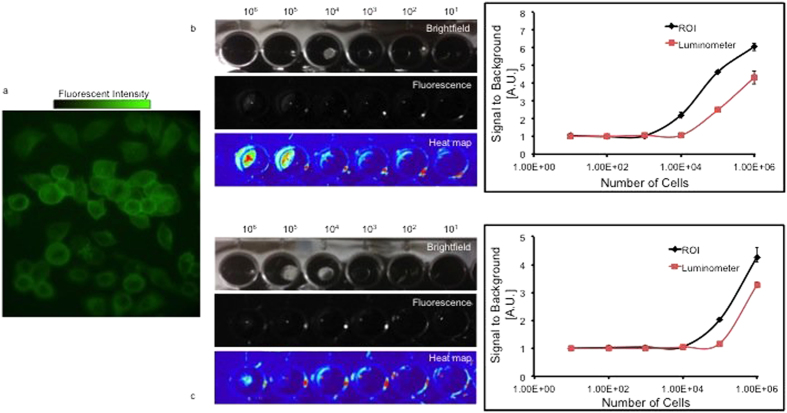
(**a**) KB cells incubated with 18.4 μM EC-17 under 200× magnification fluoresce upon excitation by 490 nm light. (**b**) KB cells incubated with 18.4 μM EC-17. Black wells containing increasing logarithmic values of cells were imaged, and the signal was quantified using the luminometer and ROI software. The pseudocolor map shows decreasing areas of detectable fluorescence, and some glare is present in all wells. Y-axis is measured in arbitrary units. Error bars are reported as standard deviations (STD). (**c**) RCC10 cells incubated 18.4 μM EC-17. Black wells containing increasing logarithmic values of cells were imaged, and the signal was quantified using the luminometer and ROI software. Y-axis is measured in arbitrary units. Error bars are reported as standard deviations (STD).

In order to further evaluate each system, we repeated our studies *in vitro* using tumor cells treated with EC-17. KB or RCC10 cells were incubated with 18.4 μM EC-17 for 45 minutes, washed and plated 1 cell/3.3 × 10^−3^m^2^ −1 cell/3.3 × 10^−9^m^2^ in 96 well plates (3.3 × 10^−3^m^2^/well). The culture plates were then imaged using microscopy, digital imaging and the luminometer (Fig. 4a–c) SBRs were generated using background signal from wells with non-fluorescent tumor cells. In a 96-well plate, the smallest quantity of cells the luminometer and optical imaging could detect was similar and was between 10^4^ and 10^5^ cells. Both quantification systems detected a more intense signal from KB cells (Fig. 4b) than RCC10 cells (Fig. 4c), reflecting greater uptake of the probe in the folate-receptor positive KB cells. At 1 million KB tumor cells/well, optical imaging and the luminometer had SBR ratios of 6.1 ± 0.2 and 4.3 ± 0.4 (p = 0.001), respectively (n = 3). At 1 million RCC10 tumor cells/well ROI and the luminometer had SBR ratios of 4.3 ± 0.3 and 3.3 ± 0.1 (p = 0.004), respectively (n = 3).

The digital ROI analysis was more sensitive than the luminometer for identifying trace levels of fluorescence from small quantities of fluorescent tumor cells. The digital capture software identified 10^4^ cells/well, whereas the luminometer was sensitive to 10^5^ cells/well. Furthermore, when we compared the SBRs for both devices at 10^6^ cells/well, the fluorescent signal was higher with the digital ROI method. The SBR generated for 10^6^ KB cells/well co-cultured in EC-17 was larger when using ROI than when using the luminometer (6.1 vs. 4.3) or the ROI software (4.3 vs. 3.3).

### Quantification of tumor fluorescence in murine models

**Figure 5:**
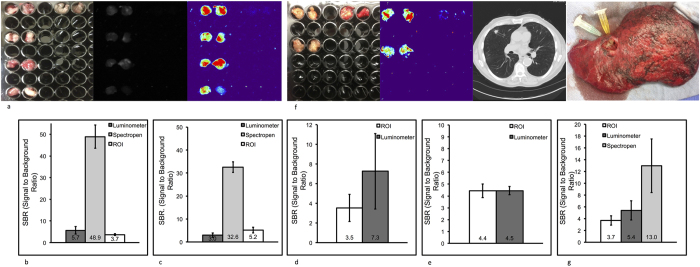
(**a**) Brightfield, fluorescent, and pseudocolor images of LLC flank tumors from BL6 mice injected with ICG. (**b**) Signal-to-background ratio of TC1 murine flank tumors imaged with ICG. Error bars are reported as standard deviations (STD). (**c**) Signal-to-background ratio of LLC murine flank tumors imaged with ICG. Error bars are reported as standard deviations (STD). (**d**) Signal-to-background ratio of KB murine flank tumors imaged with EC17. Error bars are reported as standard deviations (STD). (**e**) Signal-to-noise ratio of IGROV murine flank tumors imaged with EC17. Error bars are reported as standard deviations (STD). (**f**) CT scan, bisected nodule of human adenocarcinoma patient, bright field, and fluorescent,. (**g**) Signal-to-background ratio of human adenocarcinoma imaged with ICG. Error bars are reported as standard deviations (STD).

To test the quantification and sensitivity of each imaging technique *in vivo*, we established murine flank tumors and injected tumor-bearing mice IV with either EC-17 or ICG. We tested non-small cell lung cancer (TC1, LLC), cervical carcinoma (KB) and ovarian (IGROV) cell lines. NOD.Cg-*Pr*kdcscid *Il2rg*^*tm1Wjl*^/SzJ mice were injected with KB or IGROV cells into the right flank, and C57bl/6 mice were injected with TC1 or LLC cells into the right flank. The tumors reached a mean volume of 500 mm^3^ after about 3 weeks. The mice were injected with either 0.1 mg/kg of EC-17 or 5 mg/kg of ICG IV and the tumors were resected. The tumors were cut into approximately 0.5 cm^3^ portions and placed in a black 96-well plate for fluorescence imaging (Fig. 5a). Muscle adjacent to the tumor was used as the negative control (top row, right). Background fluorescent readings were taken from these specimens. SBRs were generated and each measurement was repeated five times.

The TC1 tumors generated the highest SBR reading (49.9 ±8.7, n = 8) measured when ICG fluorescence was quantified with the spectrometer (Fig. 5b). The luminometer was more sensitive to small amounts of tumor fluorescence when compared to ROI analysis but less sensitive than spectroscopy. The LLC cell line was the one notable exception where digital imaging had a higher SBR than the luminometer (5.2 ± 1.5 vs. 3.0 ± 1.3, p = 0.003, n = 8) (Fig. 5c). The KB cell line (Fig. 5d) produced a tumor with a SBR significantly brighter with the luminometer than with the digital analysis software (7.3 ± 3.9 vs. 3.5 ± 1.4, p = 0.01, n = 8). The ovarian flank tumor generated similar SBRs between the two quantification techniques (Fig. 5e). The average SBR of the spectrometer, luminometer and digital analysis was 41.7 ± 11.5, 5.1 ± 1.8 and 4.1 ± 0.9 (p = 0.0001), respectively (n = 3).

Together, these data suggest that it is possible to quantify the fluorescent signal from murine tumors when the animals have been injected with EC-17 or ICG. Quantification of tumor fluorescence is highly dependent on the imaging technology and must be done in concert with an *ex vivo* standard curve. This high throughput murine model is reliable and was easy to use. Several cancer types were successfully visualized with fluorescence imaging. It is an inexpensive strategy to investigate the quantification of fluorescent tumors.

### Quantification of tumor fluorescence in human clinical trial

Lastly, to test the ability of our fluorescent technologies to detect optical contrast agents in humans, we investigated 3 human patients who were injected with ICG 24 hours prior to the removal of a primary pulmonary adenocarcinoma as part of an ongoing clinical trial^2^. A representative patient is shown in Fig. 5f.

The test patient was a 68 year old male with a 1.5 cm right upper lobe non-small cell lung cancer (Fig. 5f). The patient had no evidence of systemic disease, thus he was scheduled for surgery with intraoperative imaging assistance. The patient was injected IV with 5 mg/kg of ICG 24 hours prior to surgery. During surgery, the patient’s chest was opened and the nodule was resected (Fig. 5f). Intraoperatively, the tumor was subjectively fluorescent on the digital imaging monitor when reviewed by the surgeon. A piece of the tumor (3.6 grams, wells on left) and surrounding latissimus muscle (wells on right) were harvested for more detailed quantification of the fluorescence. The latissimus muscle was used as a background signal. SBRs were generated and each measurement was repeated in triplicate. The tumor was portioned and quantified using the luminometer, spectrometer and digital analysis (Fig. 5g).

The spectrometer generated the highest SBR (13.0 ± 4.6, n = 8) compared to the luminometer (5.4 ± 1.6, n = 8) and digital analysis (3.7 ± 0.8, n = 8). Using the standard curve of ICG generated in Fig. 3, we then attempted to quantify the concentration of the ICG in the tissue. According to our prior calculations, we estimate the [ICG] in the tumor tissue to be 5.5 × 10^−6^ M. These data indicate that the optical contrast agents may accumulate at up to 13-fold higher concentrations in tumors than healthy adjacent tissue. The spectrometer was the most sensitive at identifying fluorescence, however, the luminometer and digital analysis did produce distinguishable differences in SBR.

### Signal detection as a function of depth

**Figure 6:**
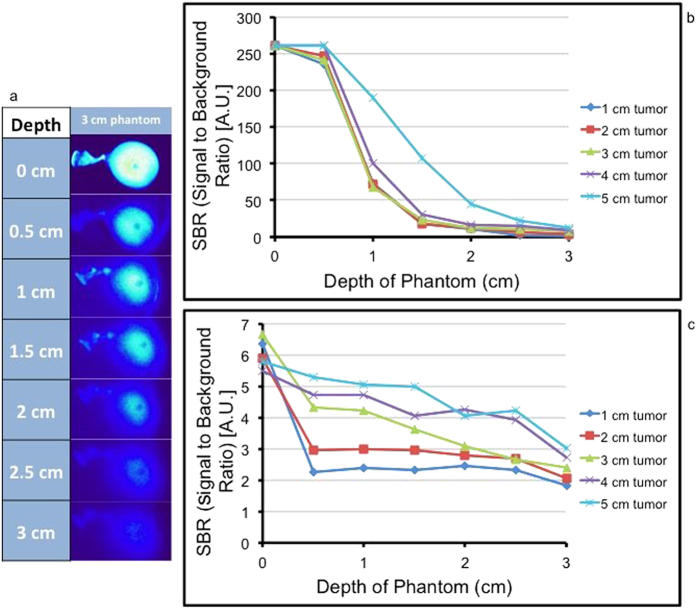
(**a**) Fluorescent images of 3 cm ICG balloon phantoms submerged under various depths of liquefied butter. The Flocam ROI SBR data was gathered from these images. (**b**) Flocam ROI SBR vs. depth of tumor. ICG phantom sizes range from 1–5 cm. (**c**) Spectropen SBR vs. depth of tumor. ICG phantom sizes ranges from 1 cm–5 cm.

One of the shortcomings of intraoperative cancer detection is the limited ability of imaging techniques to locate tumors at increasing tissue depths. In order to test the ability of each imaging technique to detect fluorescence at varying depths of penetration, we imaged ICG phantoms in a semi-solid tissue model of adipose tissue. Balloons were filled with 3.23 μM ICG until they reached 1.0, 2.0, 3.0, 4.0, and 5.0 cm in diameter. They were submerged under semi-solid butter from 0 to 3 cm at 0.5 cm intervals (Fig. 6a). The digital capture software and spectrometer were used to quantify fluorescent signal from the phantoms.

Both digital imaging ROI and the spectrometer produced higher SBRs for un-submerged phantoms compared to phantoms embedded in 3 cm of semi-solid butter (6.0 ± 0.5 vs. 2.4 ± 0.5 (p = 0.00001) and 261.5 ± 0.1 vs. 7.0 ± 3.4 (p = 0.00001), respectively (n = 3). The spectrometer generated a maximum SBR value of 261.5 and the digital analysis software generated a maximum SBR of 6.7 (Fig. 6b,c). Likewise, the 5 cm phantom consistently had the highest SBR when measured with the spectrometer, and the different sized phantoms had distinct differences. With the spectrometer, however, all phantoms smaller than 4.0 cm in diameter have similar SBRs (Fig. 6b). The 5.0 cm phantom generally had the highest signal. There was a large decrease in the SBRs for each phantom when the depth of penetration increased from 2.5 cm to 3.0 cm. There was a larger gap between the signals from different-sized tumors when compared to the spectrometer values.

Thus, we found that both the size of the phantom and depth below the surface had important effects on the measured fluorescent signal. Using the model of adipose tissue, we found that although the spectroscopy signals were amplified in comparison to the digital capture system, they both maintained the same trend.

## Discussion

Non-specific and receptor targeted fluorophores are being used in combination with various fluorescent imaging systems for intraoperative tumor visualization. This study compares three imaging modalities– spectroscopy, luminometry and digital imaging – to obtain quantitative data from tumor fluorescence during cancer surgery (Table 1). We found that all three imaging devices are clinically feasible and provide useful information. For detecting both small numbers of cancer cells and detecting tumors deeper in tissues (up to 3 cm), spectroscopy is more sensitive and has superior resolution than luminometry and digital imaging. With increasing residual disease >106 cells), spectroscopy has no advantage over digital imaging because the magnitude of fluorescence from the cancer cells overwhelms the resolution of spectroscopy, and this approach is slow and time-consuming. In these settings, digital imaging has substantial advantages. It provides real-time, high-resolution images to the surgeon, which allows for intraoperative decisions. Although it is not as sensitive for identifying small quantities of disease, it may be the most useful approach for routine cancer operations. Ultimately, an approach using digital imaging to survey a large region and then spectroscopy to verify targeted areas of interest may be the best combination.

In this study, we tested our 3 imaging systems using 2 commercially used fluorophores: ICG and EC-17. EC-17’s extinction coefficient (7.5 × 10^4^ M^−1^cm^−1^) is two-fold that of ICG (4 × 10^4^ M^−1^cm^−**1**^), thus it was useful to test each system with two different fluorophores. While EC-17 is a receptor-targeted fluorophore, ICG does not specifically target cancer cells. ICG accumulates by the enhanced permeability and retention (EPR) effect in solid cancers. When injected systemically, ICG passively accumulates in tumors due to wide blood vessel fenestrations and defective endothelial cells^29^. The ICG is then retained due to its molecular size, shape, differences in tumor oncotic pressure and poor lymphatic angiogenesis^30^,^31^,^32^. Spectroscopy, as predicted, is the most sensitive at detecting areas of minimal fluorescence. We could detect 0.1 μM ICG *in vitro*, whereas the lowest threshold for digital imaging was closer to 1 μM. Furthermore, spectroscopy was superior at fine-point discrimination of small increases in fluorescence. Of note, in our murine model, we noticed that a standard curve was useful in estimating the relative fluorescence of tumor tissues and normal background organs. Thus, in clinical situations where a surgeon may be inspecting a close margin, spectroscopy provides the ideal approach for detailed interrogation to detect residual cancer cells. Molecular imaging can detect up to 50% more residual tumor deposits than traditional margin detection, generating a 50% increased recurrence-free survival rate^33^,^34^. Moreover, multiple-organ recurrence is less likely to occur with fluorescence-guided surgery^35^. Our study confirms the benefit of fluorescent-guided tumor resection over standard macroscopic resection, in which up to 85% residual tumor deposits are detected with fluorescent imaging versus only 9% for residual nodules captured without fluorescence imaging^24^.

We also considered the ability of each imaging technology to identify small (1 cm) and large (5 cm) ICG tumor phantoms located deeper in simulated tissue. With increasing tumor depth and tissue density, scattering and absorption limit the fluorescence that can be measured at the surface. Again, we found spectroscopy was capable of identifying low levels of fluorescence from 2 cm tumors as far as 5 cm from the surface of the phantom. Digital imaging, on the other hand, could not measure detectable signal beyond 2.5 cm below the phantom surface. For bigger tumors, however, both spectroscopy and digital imaging could identify the location of the fluorescence. Clinically, spectroscopy may have greater value in the localization of small tumors in solid organs (e.g. subcentimeter pulmonary nodules, hepatic colorectal metastases) that may be precarious to cut into due to bleeding or loss of tissue. For larger tumors that have a high fluoroescence, digital imaging may be sufficient.

Finally, with regards to ease of use, the digital imaging approach was significantly better than spectroscopy or the luminometer. The ability of a surgeon to visualize the fluorescence in a wide field of view provides rapid interrogation of an entire organ surface. We found we could inspect up to 100 mm^2^ of an organ surface within an integration time of 1 second. Spectroscopy, on the other hand, only allowed us to examine 1 mm^2^ per scan and this limits its practical application.

There are a number of factors that must be taken into account in the interpretation of the study. First, we selected three representative imaging devices: spectroscopy, luminometer and digital imaging. Each device has significantly different light sources (lasers versus light emitting diodes), illumination methods, detectors and detection bandwidths. The spectroscopic device is a hand-held device that, to our knowledge, is the only one available for clinical use. The luminometer is a mid-range device and more expensive machines will have greater resolution power. Digital imaging charged coupled devices also span a wide range of resolution. We constructed a device in the mid-price range, but the high-resolution cameras that are 3 to 5 fold more expensive may begin to match spectroscopic devices.

The optical properties of the fluorophores must also be considered. Longer wavelength NIR fluorophores will be better observed in tissues as they are less subject to light scattering and absorption and out of the range of tissue autofluorescence. The brightness of the probe, related to both the extinction coefficient and the quantum yield is also important. Finally, we draw attention to the heterogeneity of human tumors. Due to complex tumor heterogeneity and variable tumor perfusion and drainage, the uptake of contrast agent is likely to be the major limiting factor in intraoperative imaging. Thus, it is challenging to draw broad conclusions about devices without controlling the model, which is not possible with human tumors. Further human trials will be needed to explore these issues.

Based on our observations, spectroscopy and digital imaging are likely to be the optimal approach to intraoperative imaging, and more useful than luminometry. The tumors need to be imaged in a dark environment under special conditions, which is often impractical in the confines of the operating room. Future technologies that combine spectroscopy and digital imaging will have major advantages. This approach will allow for detailed quantitative information about the fluorescence in and around the tumor. It will also provide a wide field of view and practical information to the surgeon for ease of use and clinical utility. The most useful strategy will be to use digital imaging to scan large regions of body cavity and the wound. Then, for more detailed analysis of margins and lymph nodes, it will be necessary to utilize the spectroscopic device.

## Additional Information

**How to cite this article**: Judy, R. P. *et al.* Quantification of tumor fluorescence during intraoperative optical cancer imaging. *Sci. Rep.* **5**, 16208; doi: 10.1038/srep16208 (2015).

## Figures and Tables

**Table 1:** Imaging modality comparison.

Imaging Approach	Advantages	Disadvantages	Considerations	Suggested Uses
Spectroscopy	Most sensitive to small quantities of disease. Can identify minimal disease up to 3 cm depth of penetration.	Limited field of view.	Provides 1800 data points per reading, thus data processing is time consuming.	Identifying small areas of residual disease.
Luminometry	Precise reproducible measurements.	Cannot be used *in vivo.*	Can only evaluate small regions of tissue.	Not useful for clinical application.
Digital imaging	Wide field of view.	Subject to user bias.	Sensitivity depends on quality of charge coupled device.	Useful for broad exploration of the wound and body cavity.
